# Complete Genome Sequence of Klebsiella pneumoniae Podophage Pone

**DOI:** 10.1128/MRA.01405-20

**Published:** 2021-05-27

**Authors:** Johnathan Lo, Lauren Lessor, James Clark, Tram Le, Jason J. Gill, Mei Liu

**Affiliations:** a Center for Phage Technology, Texas A&M University, College Station, Texas, USA; Loyola University Chicago

## Abstract

Klebsiella pneumoniae is a Gram-negative pathogen that has become increasingly antibiotic resistant. Phage therapy is potentially a useful approach to controlling this pathogen. Here, we present the genome sequence of the phiKMV-like K. pneumoniae podophage Pone.

## ANNOUNCEMENT

Klebsiella pneumoniae is a nonmotile Gram-negative bacillus found in the soil, mouth, skin, and gastrointestinal tract. It has received attention in recent years as a panresistant, nosocomial pathogen (1), particularly the highly resistant carbapenemase-producing strains (2, 3). They are also known to carry a wide array of other antibiotic resistance genes and are reservoirs for drug resistance elements resulting in endemic antibiotic resistance among the *Enterobacteriaceae* (4). Phage therapy is one promising solution to these panresistant pathogens, and hence it is useful to characterize the phages of K. pneumoniae to evaluate their therapeutic potential. Here, we present the genome sequence of K. pneumoniae phage Pone.

Bacteriophage Pone was isolated from wastewater collected in College Station, TX, based on its ability to form plaques on the clinical isolate K. pneumoniae 43421 (GenBank accession no. NZ_NDDH00000000), using the soft-agar overlay method, and phage purification was carried out by picking and replating the isolated plaques for several rounds on soft-agar overlay seeded with the host strain as described previously (5). Phage and host bacteria were aerobically cultured on Trypticase soy broth or agar at 37°C. The morphology was determined to be podophage by negative staining the sample with 2% (wt/vol) uranyl acetate (6) and viewing it through transmission electron microscopy at the Texas A&M Microscopy and Imaging Center. Phage DNA was extracted using the Promega Wizard DNA extraction system following a modified protocol as previously described (7), and a DNA library was prepared with average 300-bp inserts using the TruSeq Nano kit (Illumina). Samples were sequenced with 300-cycle chemistry on an Illumina iSeq 100 platform. Read quality control was conducted using FastQC (www.bioinformatics.babraham.ac.uk/projects/fastqc) on the 775,946 raw reads, and the genome was assembled from these reads using SPAdes v3.5.0 (8), resulting in a single contig with 367.6-fold coverage. The contig was closed and verified using PCR and Sanger sequencing with the forward primer GTGCCTAGCGCCAAAAAGAG and the reverse primer CACTGGACAGGCACTAGAGG. Structural annotation was conducted using GLIMMER v3 (9) and MetaGeneAnnotator v1.0 (10), with manual corrections. ARAGORN v2.36 (11) was used to detect potential tRNAs. Functional annotation was conducted using sequence similarity searches from BLAST v2.9.0 (12), conserved domain searches from InterProScan v5.33 (13) and HHPred (14), and membrane topology predictions from TMHMM v2.0 (15). BLAST searches were conducted against the NCBI nonredundant (nr) and Swiss-Prot databases (16). Genomic comparisons were conducted using progressiveMauve v2.4 (17). All analyses were conducted via the CPT Galaxy and Web Apollo interfaces (18–20) with default settings.

Phage Pone is a 44,346-bp podophage, with terminal repeats predicted using PhageTerm (21). The precise repeat boundaries could not be identified. Its genome contains 53.8% G+C content and 56 protein-coding genes at a 94% overall coding density. Of the 56 protein-coding genes, 36 were assigned putative functions. Comparative analysis of the genome shows a high degree of identity with phiKMV (GenBank accession no. NC_005045). Phage Pone shares 19 out of 48 phiKMV proteins according to a BLASTp comparison (E < 0.001) and 30% similarity at the DNA level with phiKMV according to a progressiveMauve analysis. The arrangement of genes and the genome size of phage Pone are consistent with a phiKMV-like phage (22), with the notable exception of the first 7 kb, which encodes an array of hypothetical proteins that contain no detectable similarity to any genes of known function, and a second region at nucleotide position 14097 to 16649 containing hypothetical proteins with transmembrane domains and secretion signals. This likely reflects the specialization of phage Pone for host takeover and infection processes.

### Data availability.

The genome sequence of phage Pone was deposited under GenBank accession no. MT701589 and BioSample accession no. SAMN14609640. The BioProject accession number is PRJNA222858, and the SRA accession number is SRR11558349.
